# Prevalence of tick-borne pathogens in ticks from migratory birds in republic of Moldova

**DOI:** 10.1186/1756-3305-7-S1-P4

**Published:** 2014-04-01

**Authors:** AC Morozov, AA Tischenkov, AA Proka, C Silaghi, IK Toderas, AA Movila, S Poppert

**Affiliations:** 1 Institute of Zoology, Academy of Science of Moldova, Chiûinău, Republic of Moldova; 2 Dniester State University named T.G. Shevchenko, Tiraspol, DMR, Republic of Moldova; 3 Comparative Tropical Medicine and Parasitology, Ludwig-Maximilians-Universität, Munich, Germany; 4 Bernhard Nocht Institute For Tropical Medicine, Hamburg, Germany; 5 Justus Liebig University, Giessen, Germany

## 

Migratory birds are often implicated in transporting ticks, which can carry pathogenic agents of several human and domestic animals diseases. By themselves ticks are not highly mobile, and the only ways to expand the habitat are hosts. Birds, due to the ability to fly, have the greatest influence on the resettlement of ticks. As was documented by many authors birds can transport ticks harboring *Borrelia *spp., *Babesia *spp., *Anaplasma *spp. and *Rickettsia *spp. throughout many parts of Europe.

Migratory birds were caught in ornithological nets from March 2012 till October 2013 at natural forest reservations (Yagorlik, Prutul de Jos, Padurea Domneasca), and urbanocenoses of Chisinau and suburbs. All birds were identified to species level, and their sex. Birds were placed in bags made of thick fabric, right after birds were examined in the camp for the presence of ticks. After the examination, birds were released without being harmed. Tick species were detached from birds and stored individually in 70% alcohol. Total DNA was extracted using QIAamp DNA Mini Kit. Specific PCRs were carried using DNA to detect *Borrelia*, *Anaplasma*, *Rickettsia, Neoehrlichia, Brucella *species.

Total of 131 ticks relating to the 3 ticks species (*Ixodes ricinus *n = 124, *I. frontalis *n = 6, *Haemaphysalis punctata *n =1) were collected from 45, tick-infested birds relating to the 7 avian species. The most common host was *Turdus merula*. From 131 ticks in extracted DNA of 77 one or more of the following pathogens was detected: 25 ticks were positive for *Anaplasma phagocytophilum*, 18 were positive for *Borrelia garinii*, 1 was positive for *B. lusitaniae *, 1 for *B. valaisiana*, 9 for *Rickettsia monacensis*, 2 ticks were positive for *Rickettsia helvetica*, 1 for *Rickettsia typhi*, 2 for *Neoehrlichia mikurensis*, 2 for *Babesia microti *and 2 for *Borrelia miyamotoi*. These included 12 cases with a mix of the two pathogens and 2 cases with three pathogens.

The presence of the above pathogens in ticks collected from passerine birds corroborates the role of these vertebrates in the epidemiology and dispersion of tick-borne diseases. Some of the birds are considered as migratory or partial migratory. In addition these birds share the ecologic niche with the others migratory birds which migrate for long distances from Africa to the Europe. Our data suggest the involvement of ticks parasitic on birds in the cycle of human and cattle tick-borne diseases.

